# Supplementary material for: Benthic ecosystem cascade effects in Antarctica using Bayesian network inference
Source: Commun Biol. 2020 Oct 16;3:582. doi: 10.1038/s42003-020-01310-8 (PMC7567847; doi:10.1038/s42003-020-01310-8)
Supplement: Supplementary file 1 — Supplementary Information [file 42003_2020_1310_MOESM1_ESM.pdf]

Benthic ecosystem cascade effects in Antarctica using Bayesian network inference

Authors: Emily G. Mitchell*[1], Rowan J. Whittle[2] & Huw J. Griffiths[2].

Affiliations:

[1] Department of Zoology, University of Cambridge, Downing St, Cambridge, CB2 3EJ. E-mail: ek338@cam.ac.uk

[2] British Antarctic Survey, High Cross, Madingley Road, Cambridge, CB3 0ET

*Correspondence to: ek338@cam.ac.uk.

Supplementary Tables

Depth		Region		Substrate	
500	181	N	70	Silt	204
750	205	NE	151	Rocky	210
900	141	NW	146	Gravel	74
		S	70	Drop stone	5
		W	90	Boulders	24
				Biogenic	10

Supplementary Table 1.

Table of count data for the physical variables for the fine-scales analyses. Left hand side column within each physical variable are the different groups, and the numbers are the abundance of each group. Depth is measured in meters. The substrate Biogenic refers to those organisms which are living off other organisms either alive or dead.

Abundance group	Percent Encrusting	VME Unknown	Arthropod	Cnidarian	Bryozoan	Echinoderm	Porifera
Zero	0	395	376	277	348	113	319
Low	281	131	108	145	95	224	119
High	246	1	43	105	84	190	89

Supplementary Table 2.

Count data for the taxa variables included in the fine-scale analyses. Total species counts for the areas studied, the number of abundant species are those which occurred in >33% of grid-cells.

Depth		Region		Substrate	
500	10	N	3	Silt	12
750	8	NE	5	Rocky	8
900	6	NW	9	Gravel	4
		S	4		
		W	3		

Supplementary Table 3.

Count data for the physical taxa variables included in the large-scale analyses. Left hand side column within each physical variable are the different groups, and the numbers are the abundance of each group. Depth is measured in meters.

	Annelid	Arthropod	Cnidarian	Echinoderm	Mollusc	Bryozoan	Actinopteri	Ophiuroidea	Euryalida	Porifera	VME unknown	Percent encrusting
Zero	14	4	2	3	5	5	11	1	15	4	6	0
Low	6	9	10	9	8	9	8	10	4	9	8	10
High	1	8	9	9	8	9	2	10	2	8	7	3
Higher												7
Highest												1

Supplementary Table 4.

Count data for the taxa variables included in the large-scale analyses. Total species counts for the areas studied, the number of abundant species are those which occurred in >33% of grid-cells. Only percent encrusting had five different discrete groups.

Taxon	Median	Max
VME unknown	4	132
Percent encrusting (mean)	8.45	50
Bryozoan	2	12
Porifera	2	50
Echinoderm	3	75
Arthropod	1	9

Supplementary Table 5.

Median and maximum values for the fine-scale data. Median values are used to define the boundary between Low and High counts apart from Percent encrusting which is done using quartiles.

Measure	VME.unknown	Percent encrusting	Annelid	Arthropod	Cnidarian	Eechinoderm	Mollusc	Bryozoan	Actinopteri	Porifera	Ophiuroidea	Euryalida
Mean	239.19	6.05	2.43	12.11	73.55	145.85	5.63	32.31	1.18	39.50	116.60	11.13
Median	47.5	5	2	12	18.5	81.5	2.5	19.5	1	26	38	3
Max	2110	16.16	8	32	420	1116	24	124	2	174	1096	34

Supplementary Table 6.

Mean, median and maximum values for the large-scale data. Median values are used to define the boundary between Low and High counts apart from Percent encrusting which is done using quartiles.

Abundance state	VME Unknown	Porifera	Mollusca	Euryalida	Ophiuroidae	Arthopoda	Echinodermeta	Cnidaria	Bryozoa
Zero	28.55%	25.82%	39.05%	48.95%	17.68%	-13.59%	39.03%	37.37%	44.44%
Low	-21.48%	-19.05%	-33.08%	-35.79%	-13.05%	13.13%	-31.37%	-30.11%	-22.22%
High	-7.07%	-6.77%	-5.97%	-13.17%	-4.63%	0.46%	-7.66%	-7.25%	-22.22%

Supplementary Table 7.

Percentage change in abundance state when the substrate type is changed from Silt to Rocky. Percentages show the relative increase or decrease in the probability the taxon given in the two row of being in that state given by the first column.

Abundance state	VME Unknown	Porifera	Mollusca	Euryalida	Ophiuroidae	Arthopoda	Echinodermeta	Cnidaria	Bryozoa
Zero	-75.41%	-74.99%	-44.34%	-26.73%	-64.22%	2.76%	-47.56%	-44.05%	-100.00%
Low	64.04%	62.95%	30.47%	26.90%	53.33%	-4.37%	38.46%	35.54%	75.00%
High	11.37%	12.04%	13.87%	-0.17%	10.88%	1.61%	9.10%	8.51%	25.00%

Supplementary Table 8.

Percentage change in abundance state when the substrate type is changed from Silt to Gravel. Percentages show the relative increase or decrease in the probability the taxon given in the two row of being in that state given by the first column.

Abundance state	VME Unknown	Porifera	Mollusca	Euryalida	Ophiuroidae	Arthopoda	Echinodermeta	Cnidaria	Bryozoa
Zero	-46.85%	-49.17%	-5.29%	22.22%	-46.54%	-10.83%	-8.53%	-6.68%	-55.56%
Low	42.56%	43.90%	-2.61%	-8.89%	40.29%	8.76%	7.09%	5.43%	52.78%
High	4.29%	5.27%	7.90%	-13.33%	6.25%	2.07%	1.44%	1.25%	2.78%

Supplementary Table 9.

Percentage change in abundance state when the substrate type is changed from Rocky to Gravel. Percentages show the relative increase or decrease in the probability the taxon given in the two row of being in that state given by the first column.
